# Influence of the CYP2D6 Isoenzyme in Patients Treated with Venlafaxine for Major Depressive Disorder: Clinical and Economic Consequences

**DOI:** 10.1371/journal.pone.0090453

**Published:** 2014-11-04

**Authors:** Antoni Sicras-Mainar, Pablo Guijarro, Beatriz Armada, Milagrosa Blanca-Tamayo, Ruth Navarro-Artieda

**Affiliations:** 1 Directorate of Planning, Badalona Serveis Assistencials, S.A., Badalona, Barcelona, Spain; 2 Pfizer, Madrid, Spain; 3 Department of Psychiatry, Badalona Serveis Assistencials, S.A., Badalona, Barcelona, Spain; 4 Medical Documentation, Hospital Germans Trias i Pujol, Badalona, Barcelona, Spain; Hospital Nacional de Parapléjicos - SESCAM, Spain

## Abstract

**Background:**

Antidepressant drugs are the mainstay of drug therapy for sustained remission of symptoms. However, the clinical results are not encouraging. This lack of response could be due, among other causes, to factors that alter the metabolism of the antidepressant drug. Objective: to evaluate the impact of concomitant administration of CYP2D6 inhibitors or substrates on the efficacy, tolerability and costs of patients treated with venlafaxine for major depressive disorder in clinical practice.

**Methods:**

We designed an observational study using the medical records of outpatients. Subjects aged ≥18 years who started taking venlafaxine during 2008–2010 were included. Three study groups were considered: no combinations (reference), venlafaxine-substrate, and venlafaxine-inhibitor. The follow-up period was 12 months. The main variables were: demographic data, comorbidity, remission (Hamilton <7), response to treatment, adverse events and costs. The statistical analysis included logistic regression models and ANCOVA, with p values <0.05 considered significant.

**Results:**

A total of 1,115 subjects were recruited. The mean age was 61.7 years and 75.1% were female. Approximately 33.3% (95% CI: 30.5 to 36.1) were receiving some kind of drug combination (venlafaxine-substrate: 23.0%, and venlafaxine-inhibitor: 10.3%). Compared with the venlafaxine-substrate and venlafaxine-inhibitor groups, patients not taking concomitant drugs had a better response to therapy (49.1% *vs.* 39.9% and 34.3%, p<0.01), greater remission of symptoms (59.9% *vs.* 50.2% and 43.8%, p<0.001), fewer adverse events (1.9% *vs.* 7.0% and 6.1%, p<0.05) and a lower mean adjusted cost (€2,881.7 vs. €4,963.3 and €7,389.1, p<0.001), respectively. All cost components showed these differences.

**Conclusions:**

The patients treated with venlafaxine alone showed a better response to anti-depressant treatment, greater remission of symptoms, a lower incidence of adverse events and lower healthcare costs.

## Introduction

Depression is a major public health issue due to its high frequency, disability and mortality rate, and its impact on healthcare resource use and individual loss of productivity [1], [2]. According to the ESEMeD study, major depressive disorder (MDD) has an estimated yearly prevalence of 3.9% and a lifetime prevalence of 10.5% in Spain [3] and 14% of patients seen in primary care in Spain have MDD [4].

Antidepressants (AD) are the mainstay of drug therapy for sustained remission of symptoms [5]. However, although a wide range of AD is available, clinical results are not encouraging. About 38% of patients do not respond to AD treatment, and 54% do not achieve complete remission of symptoms [6]. This lack of response or adverse effects at generally effective and well tolerated doses could be due, among other causes, to factors that alter the metabolism of antidepressant drugs [7].

Many AD in current use are substrates of the cytochrome P450 2D6 enzyme (CYP2D6). This coenzyme has a high degree of polymorphism, resulting in a variety of phenotypes between individuals [8]. Approximately 7–10% of Caucasians are poor metabolizers of CYP2D6 [9], [10]. In these individuals, drugs subject to first-pass hepatic metabolism by CYP2D6 have a higher exposure of the parent compound than expected and fewer active metabolites. Furthermore, co-administration of a CYP2D6 inhibitor may also affect the pharmacokinetics of AD in which the metabolism is dependent on CYP2D6 activity (an effect known as phenotypic conversion) [11]. These two phenomena have been associated with lower efficacy and tolerability of AD, depending on the characteristics of the drug [11].

Venlafaxine (VEN) is an antidepressant belonging to the group of serotonin and norepinephrine reuptake inhibitors. According to *in vitro* data, venlafaxine is metabolised primarily by CYP2D6 and, to a lesser extent, by CYP3A4, CYP2C19 and CYP1A2. Given its pharmacodynamic activity, O-desmethylvenlafaxine (ODV) is the main clinically-relevant metabolite and is comparable to the parent compound. Both venlafaxine and ODV are chiral compounds, with a differing pharmacokinetic profile depending on their permeability in the brain (P-glycoprotein) [12].

Studies have shown that venlafaxine has a reduced antidepressant effect in patients with a slow CYP2D6 metabolizing phenotype, which is determined by the ODV/VEN ratio [8], [13]. Another recent study reported a prevalence of phenotypic conversion of CYP2D6 of 24% in MDD patients treated with venlafaxine. Phenotypic conversion was associated with the concomitant use of other drugs, especially CYP2D6 inhibitors and substrates [11]. However, to date, no study has evaluated the results of these trials in real life situations. The aim of this study was to evaluate the impact of concomitant administration of CYP2D6 substrates or inhibitors on the efficacy, safety and resource consumption of patients treated with venlafaxine for MDD in daily clinical practice.

## Materials and Methods

### Design and study population

We conducted a multicentre, observational study through retrospective review of medical records (computerised databases) of outpatients from six primary care centres (Apenins-Montigalà, Morera-Pomar, Montgat-Tiana, Nova Lloreda, Martí-Julià and El Progrés) and the Hospital Municipal de Badalona, all managed by Badalona Serveis Assistencials, S.A., and hospital discharge reports from the Hospital Germans Trias i Pujol, Badalona. The population assigned to these centres is mostly urban, with middle-low socioeconomic status.

Data confidentiality was respected pursuant to the Personal Data Protection Act (Law 15/1999, of 13 December), with encoding of personal data. The study was classified by the Spanish Agency of Medicines and Medical Devices as a Post-Authorisation Study - Other Designs and was subsequently approved by the Independent Ethics Committee of the Hospital Clínic of Barcelona. ORCID: 0000-0002-8975-7303. This study was not interventional. In order to conduct this retrospective study, data was collected anonymously and encrypted, according to the Spanish Organic Law on Personal Data Protection. Patients gave written consent that their information be stored in the hospital database and used for research.

### Inclusion and exclusion criteria

Inclusion criteria were: a) age ≥ 18 years, b) patients who presented with an episode of MDD (according to the criteria of the International Classification of Primary Care [ICPC-2] [14] and/or the Diagnostic and Statistical Manual of Mental Disorders - Text Revision [DSM-IV-TR]) [15] and were treated with venlafaxine between 1/1/2008 and 31/12/2010, c) that the prescription complied with the minimum criteria for correct treatment (at least 8 weeks of AD treatment after the first prescription), d) patients who were included in the chronic medication programme (with a record of the daily dose, time interval and duration of each treatment), and e) with a clinical follow-up of ≥12 months. Exclusion criteria were: patients transferred to other primary care centres, death, and patients transferred to other regions.

### Study groups

We divided patients into three study groups: a) patients who started treatment with venlafaxine and did not receive substrates or inhibitors, b) patients who started treatment with venlafaxine and received substrates (venlafaxine-substrate group), and c) patients who started treatment with venlafaxine and received inhibitors (venlafaxine-inhibitor group). The potential impact of CYP2D6 inhibitors and substrates was analysed individually and by therapeutic group. Follow-up of the main study variables was for 12 months from start of treatment: frequency of combinations, remission, response to treatment, safety (adverse events) and calculation of healthcare and non-healthcare costs.

### Definition of MDD, remission and response to treatment

The Hamilton scale was used to diagnose the severity of MDD (intensity of depressive symptoms; Hamilton scale ≥ 18 points) in adults [16]. A patient was considered to be in symptomatic remission of MDD when the total Hamilton depression scale score was ≤ 7 points after at least 8 weeks of correct AD treatment [17]. No remission was defined as the presence of residual symptoms after dose- and time-appropriate pharmacological treatment. Hamilton scale evaluations were performed by the doctors or nursing staff caring for the patient. Response to treatment was defined as a reduction of ≥ 50% in the initial Hamilton scale score, partial response as a reduction of 25–49% and non-response as a reduction of <25% [18]. Hamilton scale data that could not be obtained from the computerised records were obtained by comprehensive review of the medical history.

### Sociodemographic and comorbidity data

The main variables studied were: age (continuous and ranges) and gender, as well as the following comorbidities obtained from the ICPC-2 [14]: hypertension (K86, K87), diabetes mellitus (T89, T90), dyslipidaemia (T93), obesity (T82), smoking (P17), alcoholism (P15, P16), all types of organ failure (heart, liver and kidney), ischaemic heart disease (codes: K74, K76, K75), stroke (K90, K91, K93), chronic obstructive pulmonary disease (R95, chronic airflow obstruction), bronchial asthma (R96), dementia or memory disorders (P70, P20), neurological diseases: Parkinson's disease (N87), epilepsy (N88), multiple sclerosis (N86) and other neurological diseases (N99), and malignancies (all types, A79, B72-75, D74-78, F75, H75, K72, L71, L97, N74-76, R84-86, T71-73, U75-79, W72-73, X75-81, Y77-79).

The general comorbidity summary variables used for each patient treated were: a) the Charlson comorbidity index [19] and b) the individual causality index, obtained from the Adjusted Clinical Groups (ACG), a patient classification system based on iso-resource use [20]. The Grouper ACG Case-mix system algorithm consists of a series of consecutive steps which provide 106 mutually-exclusive ACG groups, one for each patient seen. The ACG application provides resource utilisation bands (RUBs) for each patient, according to general morbidity, in one of five mutually-exclusive categories: 1 (healthy or very low morbidity users), 2 (low morbidity), 3 (moderate morbidity), 4 (high morbidity), and 5 (very high morbidity) (Table 1). The clinical characteristics of the patients were assessed from one year before until the start of treatment with venlafaxine. All study registries were properly validated.

**Table 1:** Baseline characteristics of the study series according to study group.

Study groups	No combinations	Venlafaxine/substrate	Venlafaxine/inhibitor	Total
Number of patients	N = 744	N = 256	N = 115	N = 1,115
*Sociodemographic characteristics*				
Mean age, years	60.9 (14.1)	62.0 (14.8)	63.0 (12.4)	61.7 (14.1)
Ranges: 18–44 years	13.0%	12.5%	7.3%	11.9%
45–64 years	46.5%	39.7%	44.9%	43.8%
65–74 years	21.4%	25.0%	27.5%	23.7%
≥75 years	19.1%	22.8%	20.2%	20.6%
Gender (female)	75.4%	74.0%	76.4%	75.1%
Pensioner	62.2%	77.5%^‡^	79.6%^‡^	71.0%
*General comorbidity*				
Mean diagnosis	7.1 (3.9)	8.9 (4.3)^‡^	9.5 (4.4)^†^	8.1 (4.3)
Mean Charlson index	0.4 (0.7)	0.5 (0.6)	0.4 (0.8)	0.4 (0.7)
Mean RUB	2.9 (0.6)	3.1 (0.6)^‡^	3.1 (0.6)^†^	3.0 (0.6)
RUB-1	3.2%	1.0%	2.2%	2.2%
RUB-2	12.5%	7.6%^†^	6.7%	9.8%
RUB-3	73.2%	72.1%	70.8%	72.4%
RUB-4	9.6%	15.4%^‡^	18.0%^‡^	13.1%
RUB-5	1.5%	3.9%*	2.2%	2.5%
*Associated comorbidities*				
Hypertension	36.5%	51.7%^‡^	55.1%^‡^	45.0%
Diabetes	16.8%	21.1%	16.9%	18.4%
Dyslipidaemia	53.7%	57.8%	62.4%	56.6%
Obesity	21.2%	24.0%^†^	31.5%^†^	23.9%
Active smoker	22.3%	21.6%	16.3%	21.1%
Alcoholism	3.2%	4.4%	2.2%	3.5%
Ischaemic heart disease	6.4%	7.8%	5.6%	6.8%
Cerebrovascular accident	10.6%	14.7%	14.0%	12.6%
Cardiovascular event	15.1%	19.4%	17.4%	17.0%
Organ failure	9.5%	15.2%^‡^	21.3%^‡^	13.5%
Bronchial asthma	7.8%	6.4%	7.3%	7.2%
COPD	2.8%	3.7%	1.7%	3.0%
Neuropathies	2.1%	4.7%^‡^	1.7%	3.0%
Dementia (all types)	3.2%	11.8%^‡^	7.3%*	7.0%
Malignant neoplasms	8.9%	14.0%*	11.2%*	11.1%

Values expressed as percentage or mean (SD standard deviation), RUB: resource utilisation band, COPD: chronic obstructive pulmonary disease.

* Paired comparisons using the *No combinations* group as the reference; p value in comparisons between groups: *p<0.05, ^†^p<0.01, ^‡^p<0.001.

### Resource use and cost model

Direct healthcare costs (direct costs) were considered to be those related to medical care (medical visits, diagnostic or therapeutic applications, etc.) carried out by physicians. Non-healthcare costs (indirect costs) were those related to lost productivity (number of sick days and days of disability at work) [21]. The design of the cost system took into account the characteristics of the organisations and the degree of development of the information systems available. The product unit that formed the basis for the final calculation (during the study period) was the patient treated, and the cost was expressed as the mean cost per patient (mean unit cost)3. The rates were obtained from the cost-accounting systems at each centre, except for medications and the days of work disability. Prescriptions (acute, chronic or on demand) were quantified according to the retail price including VAT per container at the time of prescription. The costs of days of work disability were quantified according to the minimum wage (source: National Institute of Statistics) [21].

### Medications prescribed (substrates and inhibitors)

The active substances that act as CYP2D6 substrates or inhibitors were defined according to the Anatomical Therapeutic Chemical Classification System (ATC) [22] (Table 2 - adapted from Preskorn) [23]. Patients who could have been included in both the venlafaxine-substrate and venlafaxine-inhibitor groups were included in the latter. The following substrates were not included in the study as they were excluded, withdrawn or not licensed by the Spanish National Health System or were not prescribed during the study period: clozapine, thioridazine, tacrine, aprindine, encainide, mexiletine, procainamide, alprenolol, bupranolol and metoprolol. Administration of CYP2D6 substrates/inhibitors was prior to starting treatment with venlafaxine. For patients who were already receiving drug treatment prior to the administration of venlafaxine, the combined consumption of venlafaxine and substrates/inhibitors was at least 6 months; in the case of venlafaxine alone it was less than 6 months during the time of treatment.

**Table 2:** Distribution of *active substances* metabolised by the CYP2D6 genotype according to study groups.

Study groups*		Venlafaxine/substrate
Number of patients		N = 256
SUBSTRATES		
*Antidepressants*		59 (23.0%)
	Clomipramine	16 (6.3%)
	Amitriptyline	41 (16.0%)
	Nortriptyline	5 (2.0%)
	Sertraline	18 (7.0%)
	Mirtazapine	12 (4.7%)
*Antipsychotics*		42 (16.4%)
	Haloperidol	8 (3.1%)
	Quetiapine	15 (5.9%)
	Risperidone	19 (7.4%)
	Aripiprazole	15 (5.9%)
*Antiarrhythmics*		5 (2.0%)
	Propafenone	2 (0.8%)
	Flecainide	3 (1.2%)
*Oestrogens*		3 (1.2%)
	Tamoxifen	3 (1.2%)
*Beta-blockers*		43 (16.8%)
	Propranolol	10 (3.9%)
	Carvedilol	8 (3.1%)
	Timolol	25 (9.8%)
*Anti-dementia drugs*		26 (10.2%)
	Nicergoline	6 (2.3%)
	Donepezil	14 (5.5%)
	Galantamine	6 (2.3%)
*Analgesics*		70 (27.3%)
	Oxycodone	9 (3.5%)
	Codeine	20 (7.8%)
	Tramadol	41 (16.0%)

Study groups*		Venlafaxine/inhibitor
Number of patients		N = 115
INHIBITORS		
*Antidepressants*		61 (53.0%)
	Fluoxetine	18 (15.7%)
	Paroxetine	13 (11.3%)
	Bupropion	2 (1.7%)
	Duloxetine	41 (35.7%)
*Antipsychotics*		3 (2.6%)
	Chlorpromazine	1 (0.9%)
	Levomepromazine	2 (1.7%)
*Other drugs*		49 (47.8%)
	Ranitidine	25 (21.7%)
	Celecoxib	46 (40.0%)
	Amiodarone	3 (2.6%)

* Patients may consume more than one drug simultaneously.

### Safety: adverse reactions

Information was obtained from medical records of patients who discontinued treatment with venlafaxine for MDD due to an adverse reaction to the medication. The expected adverse events included: a) gastrointestinal disorders (nausea, anorexia/fatigue, xerostomia), b) nervous system disorders (headache/dizziness/dry mouth, sleep disturbances, sexual dysfunction and/or anxiety/nervousness/sweating), c) circulatory disorders (increased blood pressure, flushing/palpitations), as well as any others documented in the medical record.

### Statistical analysis

Descriptive statistics were described using means, standard deviation (SD) and 95% confidence intervals (CI). The Kolmogorov-Smirnov test was used to assess the normality of distribution. A bivariate analysis was performed using analysis of variance (ANOVA) (*a posteriori* contrasts: Scheffé), the chi-square test, Pearson's linear correlation and comparison of means for paired groups. Logistic regression was performed to obtain the variables associated with the drug combinations (venlafaxine-substrate and venlafaxine-inhibitor as dependent variables), using an entry procedure (statistic: Wald). The outpatient cost comparison was performed as recommended by Thompson and Barber [24] by analysis of covariance (ANCOVA), with gender, age, RUB and the Charlson index as covariates (method: estimated marginal means, Bonferroni adjustment). The analysis was performed using the statistical package SPSSWIN version 17.0. Statistical significance was established as p<0.05.

## Results

Of the 86,628 patients aged ≥ 18 years who were initially selected, 6.7% (N = 5,769, 95% CI: 6.5–6.9%) had a diagnosis of MDD. Of these, 1,115 patients met the inclusion/exclusion criteria and were included in the study.

Table 1 shows the baseline characteristics of patients included according to the study groups. The overall mean age was 61.7 years (SD 14.1) and 75.1% were female, with a mean of 8.1 diagnoses per patient and a RUB of 3.0. 94.8% of the study population had ≥ 1 concomitant condition along with MDD. Dyslipidaemia (56.6%), hypertension (45%) and obesity (23.9%) were the most common comorbidities.

Of the 1,115 patients, 33.3% (95% CI: 30.5 to 36.1%) were administered a combination of drugs that act on the CYP2D6 metabolic pathway: 23% (N = 256, 95% CI: 20.5 to 25.5%) venlafaxine-substrate and 10.3% (N = 115, 95% CI: 7.7–11.1%) venlafaxine-inhibitor. Patients with these combinations had higher overall comorbidity (8.9 and 9.5 *vs.* 7.1 diagnoses, p<0.01) and greater resource use (3.1 and 3.1 *vs.* 2.9 RUB/year, p<0.01) compared with patients without combinations.

In the logistic regression model, the variables that were significantly associated with the venlafaxine-substrate and venlafaxine-inhibitor groups were dementia [odds ratio (OR)  = 3.8 (95% CI: 2.1 to 6.6), p<0.001], hypertension [OR  = 2.1 (95% CI: 1.6 to 2.8), p<0.001], organ failure [OR  = 1.6 (95% CI: 1.1 to 2.4), p<0.001] and RUB [OR  = 1.5 (95%: 1.3 to 1.8), p<0.001].

Table 2 shows the ratio of substrates and inhibitors according to the study groups. The therapeutic groups that most-frequently acted as substrates were analgesics (27.3%), AD (23%), beta blockers (16.8%) and antipsychotics (16.4%). The most-frequently prescribed CYP2D6 inhibitors were AD (53%). The most-prescribed active substances were tramadol (16%), amitriptyline (16%), timolol (9.8%) and codeine (7.8%) in the venlafaxine-substrate group and celecoxib (40.0%), duloxetine (35.7%), ranitidine (21.7%) and fluoxetine (15.7%) in the venlafaxine-inhibitor group. In the venlafaxine group, the following CYP2D6 substrates were also administered concomitantly: analgesics (38.3%), antipsychotics (26.1%) and AD (17.4%), of which tramadol (29.6%), risperidone (13.0%), quetiapine (12.2%) and amitriptyline (12.2%) were the most-common active substances.

Table 3 shows the efficacy and safety of venlafaxine in clinical practice. The Hamilton scale score at start of AD was similar in the study groups (15.3 *vs.* 14.9 and 15.3 points, p = 0.347). However, compared to the venlafaxine-substrate and venlafaxine-inhibitor groups, the no-combination group had a better response to AD treatment with a reduction of ≥ 50% on the Hamilton scale (49.1% vs. 39.9% and 34.3%, p<0.01) and a greater percentage of patients with remission of symptoms (59.9 *vs.* 50.2% and 43.8%, p<0.001). Likewise, the no-combination group had fewer adverse drug reactions leading to treatment discontinuation (1.9% *vs.* 7.0% and 6.1%, p<0.05).

**Table 3:** Evaluation of efficacy and safety by study group.

Study groups	No combinations	Venlafaxine/substrate	Venlafaxine/inhibitor
Number of patients	N = 744	N = 256	N = 115
*Hamilton scale score*			
Mean baseline score	15.3 (4.3)	14.9 (4.2)	15.3 (4.2)
Mean final score	8.1 (3.3)	8.6 (3.3)*	9.1 (3.6)*
Reduction in score	−7.2	−6.3*	−6.2*
*Evaluation of initial Hamilton scale score*			
Mild depression (8–13 points)	35.5%	37.7%	37.1%
Moderate depression (14–18 points)	37.2%	40.0%	33.1%
Severe/very severe depression (≥19 points)	27.2%	22.3%	29.8%
*Evaluation of final Hamilton scale score*			
Not depressed (0–7 points)	61.6%	51.5%^†^	44.6%^†^
Mild depression (8–13 points)	25.9%	33.9%*	34.3%*
Moderate depression (14–18 points)	12.5%	14.6%*	21.1%^†^
*Response to treatment*			
≥ 50%	49.1%	39.9%^†^	34.3%^†^
25–49%	19.1%	18.6%	21.7%*
≤24%	31.8%	41.5%^†^	44.0%^†^
*Remission of symptoms*	59.9%	50.2%^‡^	43.8%^‡^
*Safety/adverse reactions*	N = 14 (1.9%)	N = 18 (7.0%)^†^	N = 7 (6.1%)*

Values expressed as percentages or means (standard deviation).

* Paired comparisons using the *No combination* group as the reference; p value in comparisons between groups: *p<0.05, ^†^p<0.01, ^‡^p<0.001.

In the 12-month follow-up, the no-combination group used fewer healthcare resources compared with the other groups: primary care visits (11.4 *vs.* 16.7 and 20.3, p<0.001), days of hospitalisation (9.5 and 14.0 *vs.* 15.7, p = 0.01), specialist visits (3.2 *vs.* 5.3 and 6.9, p<0.001), A&E visits (0.5 *vs.* 1.3 and 1.9, p<0.001) and days of temporary work disability (8.7 *vs.* 14.4 and 13.8, p<0.01).

The gross cost model is shown in Table 4. The total cost of the patients included in the study was €4.9 million, of which 67.0% were direct medical costs and 33.0% were non-healthcare costs (lost productivity), with a mean unit cost of €4,345. Of the total costs, 44.2% were attributable to primary healthcare and 22.8% to specialist care: of these, 7.9% were attributable to primary care visits and 30.9% to drug prescriptions.

**Table 4:** Gross cost model per patient/year according to study groups.

Study groups	No combinations	Venlafaxine/substrate	Venlafaxine/inhibitor
Number of patients	N = 744	N = 256	N = 115
Total healthcare costs*	1,894 (1,671)	3,585 (3,424)	4,393 (3,570)
Primary care costs*	1,356 (1,181)	2,292 (1,696)	2,747 (1,625)
Medical visits	263 (217)	387 (289)	472 (345)
Laboratory tests	109 (77)	149 (90)	159 (90)
Conventional radiology	53 (50)	59 (57)	70 (54)
Complementary tests	39 (59)	57 (77)	56 (69)
Pharmaceutical prescriptions	891 (1,044)	1,640 (1,572)	1,991 (1,449)
Specialist care costs*	538 (964)	1,293 (2,646)	1,646 (2,722)
Medical visits	148 (704)	595 (2,234)	694 (2,261)
Hospitalisation	330 (414)	549 (626)	721 (658)
A&E	60 (124)	150 (325)	231 (415)
Non-healthcare costs*	883 (3,984)	1,457 (6,130)	3,018 (9,426)
Total costs (healthcare/non-healthcare)*	2,777 (4,372)	5,041 (6,890)	7,412 (10,185)

Values expressed as means (standard deviation), in euros; CI: 95% confidence interval. * p<0.05 in all between-group comparisons. Paired comparisons using the *No combination* group as the reference; p value in comparisons between groups: *p<0.05, ^†^p<0.01, ^‡^p<0.001.

**Figure 1:**
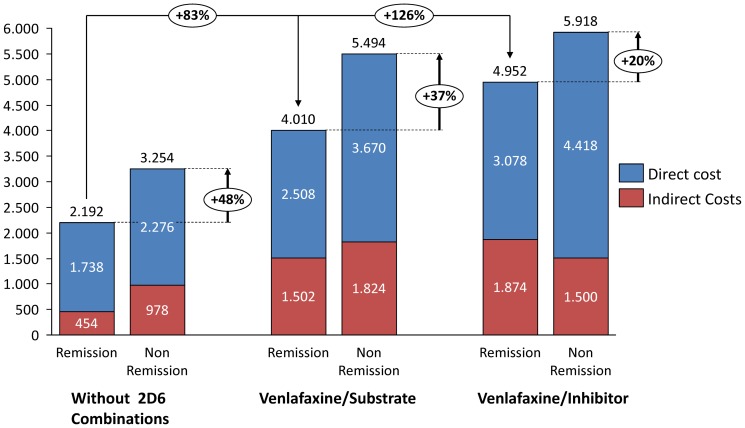
Distribution of healthcare and non-healthcare costs according to treatment response in study groups. Values expressed as means (in Euros).

With respect to the mean unit cost adjusted for covariates (ANCOVA), the total cost of patients in the no-combination, venlafaxine-substrate and venlafaxine-inhibitor groups was €2,882, €4,963 and €7,389, respectively (p<0.001). All cost components showed these differences. Additionally, differences were found in the cost associated with MDD patients according to the response to treatment in each of the study groups (Figure 1). Patients who did not achieve a response had a higher associated cost than patients who achieved remission or a better response.

## Discussion

To our knowledge, this is the first study to assess the impact of concomitant administration of CYP2D6 substrates and inhibitors on the efficacy, safety and resource use of patients treated with venlafaxine for MDD in routine clinical practice.

As reported, the metabolizing profile of CYP2D6 can cause clinically significant differences in drug efficacy when the drug in question is a substrate of CYP2D6 [8], [25]–[27]. In the case of venlafaxine, which is converted into its major active metabolite, ODV, by the action of CYP2D6, the metabolic profile can be determined using the ratio of plasma concentrations of ODV/VEN, which is higher in rapid metabolizers than in slow metabolizers. A study that pooled data from four clinical trials of patients with MDD treated with venlafaxine, found lower efficacy in slow metabolizers. Thus, there is a correlation between the CYP2D6 slow metabolizer phenotype and the response to AD treatment [8].

In addition, there are reports of phenotypic conversion in patients who did not have a slow metabolizer profile, according to genotyping, but who became slow metabolizers as a result of concomitant administration of drugs acting on the CYP2D6 pathway. The recent study by Perskorn of 865 patients treated with venlafaxine found phenotypic conversion to a slow profile in 24% of patients as a consequence of the administration of concomitant drugs, in particular CYP2D6 inhibitors or substrates [11].

The results of our study show that one in three patients with MDD treated with venlafaxine took a CYP2D6 substrate or inhibitor (23% venlafaxine-substrate and 10.3% venlafaxine-inhibitor), similar to the 27% found in the study by Preskorn [11]. In the present study, AD efficacy was greater, as shown both by the lower Hamilton scale scores and the higher percentage of patients with remission of symptoms, in patients in the no-combination group. In addition, there was a higher percentage of adverse events and higher healthcare and non-healthcare costs in patients in the venlafaxine-substrate and venlafaxine-inhibitor groups.

These findings suggest that, in patients with MDD, in whom medical and psychiatric comorbidity is fairly common and who may require various drug therapies, the pharmacokinetic properties of each medication should be taken into account to ensure that the response to and tolerability of AD treatment is not compromised.

Although this was not the objective of the study, the properties of a specific AD with respect to whether it is a CYP2D6 substrate or inhibitor may also affect the efficacy and safety of concomitant therapies. Many drugs are metabolised by CYP2D6, some of which are pro-drugs, metabolised by CYP2D6 into the active drug. These include tamoxifen, which is a selective modulator of the oestrogen receptor indicated for the treatment and prevention of breast cancer [28], which has been shown to reduce the risk of breast cancer by 50% [29]. Many antidepressants are more or less potent inhibitors of CYP2D6 and may affect the efficacy of tamoxifen by not permitting the active drug to be metabolised. A cohort study [26] of 2,430 patients found an increased risk of death related to breast cancer in women taking tamoxifen and concomitant paroxetine with a clear dose-response relationship and an association with the duration of overlap of the two treatments. Similarly, the efficacy of many opioids, such as tramadol, codeine and oxycodone is affected by the concomitant use of an AD that inhibits CYP2D6 [25], [27].

Non-detection of polymorphic variations of genotype CYP2D6 that discriminate between slow, fast or intermediate metabolizers and accuracy of diagnosis are the two most important limitations of the study. In addition, there are the limitations inherent to studies using population-based data, such as under-reporting of diseases and possible variations among health professionals in the routine use of the different clinical screening scales. All limitations derived from the retrospective nature of this study are also applicable, primarily those related with quality of data.

Likewise, in the present study, the duration of the drug combinations in each study group was not controlled and, therefore, should be considered as a limitation. Because not all patients were concomitantly taking venlafaxine and substrates/inhibitors during the whole follow-up period (12 months), we believe that this situation should be interpreted as another limitation of the study. Finally, the most severe cases of MDD and cases where the diagnosis was in doubt may not have been included, as these patients are normally treated at mental health centres.

A possible explanation for the overall results of the study, as well as a practical consequence, could therefore be that patients consuming substrates/inhibitors of CYP2D6 (in combination with venlafaxine) are less healthy, and are consequently expected to have greater use of health resources in the National Health System. Given these limitations, the results of this study show a clear relationship between reduced antidepressant efficacy of venlafaxine and the concomitant administration of CYP2D6 substrates or inhibitors in daily clinical practice. These findings suggest that, when deciding on an antidepressant therapy, the patient's other medication should be evaluated in order to avoid pharmacokinetic interactions that could reduce the efficacy of the antidepressant. Due to the high rate of comorbidity associated with MDD, which requires the use of concomitant medications, the ideal would be to have antidepressants with a simple metabolism that provided a more predictable antidepressant response, especially in patients with comorbidities and polypharmacy.
